# Local Anesthesia in Hemorrhoidal Operations and All Varieties of Minor Surgical Work

**Published:** 1901-04

**Authors:** 


### LOCAL ANESTHESIA IN HEMORRHOIDAL OPERATIONS AND ALL VARIETIES OF MINOR SURGICAL WORK.

Green, Chicago, (*Medical Times and Register*,) says his method of operating on hemorrhoidal tumors is as follows: First, the patient is instructed to take a cathartic the night before the operation, and an enema in the morning. With a saturated solution of boracic acid thoroughly cleanse the rectum, using a syringe or otherwise, and then immediately inject every tumor in sight with acestoria until each tumor is not sensitive to the prick of the needle. Sometimes it is best to use the bivalve speculum before, sometimes after, injection and sometimes not at all. It depends upon the condition and location of the piles.

With hemorrhoidal forceps, or Pean's artery forceps, pick up each tumor at its center, and turn it out. Use the clamp method when possible,—Kelsey's or Pratt's clamp. After turning the tumors slightly outward with the forceps which were left hanging to them, each by turn is clamped at its base. Then with a straight needle put in two or more stitches, as may be needed, back of clamp.

Remove the clamp and cut the tumor with straight scissors through the white line made by the middle blade of the clamp. There will be no hemorrhage if this line is followed. The stitches are now tied.

Then with hydrozone and hot water, one part of the former to five of the latter, syringe or spray the field of operation thoroughly.

The object of using hydrozone is two-fold: it is the safest and best germicide and hemostatic we have yet used. Not being a poison, and depending upon the oxygen it contains for its action, it is a safe remedy under all circumstances, both externally and internally. As a dressing we have several times used nothing, simply cleansing with hot water and hydrozone. An ideal dressing is ordinary sterilised gauze moistened with glycozone. Glycozone is anhydrous glycerine saturated with ozone, a powerful germicide and promoter of healthy granulation.

To prevent pain usually caused by the prick of the hypodermic needle, touch the point chosen for insertion with a glass pointed rod, dipped into 95 per cent. carbolic acid. To anesthetise the ear and stop earache, incline the patient's head to one side and drop into the ear about five drops of acestoria, or sufficient to fill the external meatus; also use acestoria hypodermatically in all cases where incisions or excisions are to be made, such as operations on ingrowing toe nails, removal of splinters from the flesh, opening boils, abscesses, carbuncles, and similar minor surgical procedures.

